# On Wiener polarity index of bicyclic networks

**DOI:** 10.1038/srep19066

**Published:** 2016-01-11

**Authors:** Jing Ma, Yongtang Shi, Zhen Wang, Jun Yue

**Affiliations:** 1 Center for Combinatorics and LPMC-TJKLC, Nankai University, Tianjin, 300071, China; 2 Interdisciplinary Graduate School of Engineering Sciences, Kyushu University, Kasuga-koen, Kasugashi, Fukuoka, Japan; 3 School of Mathematical Sciences, Shandong Normal University, Jinan 250014, Shandong, China

## Abstract

Complex networks are ubiquitous in biological, physical and social sciences. Network robustness research aims at finding a measure to quantify network robustness. A number of Wiener type indices have recently been incorporated as distance-based descriptors of complex networks. Wiener type indices are known to depend both on the network’s number of nodes and topology. The Wiener polarity index is also related to the cluster coefficient of networks. In this paper, based on some graph transformations, we determine the sharp upper bound of the Wiener polarity index among all bicyclic networks. These bounds help to understand the underlying quantitative graph measures in depth.

In order to decide whether a given network is robust, a way to quantitatively measure network robustness is needed. Intuitively robustness is all about back-up possibilities, or alternative paths, but it is a challenge to capture these concepts in a mathematical formula. During the past years a lot of robustness measures have been proposed^1^. Network robustness research is carried out by scientists with different backgrounds, like mathematics, physics, computer science and biology. As a result, quite a lot of different approaches to capture the robustness properties of a network have been undertaken. All of these approached are based on the analysis of the underlying graph—consisting of a set of vertices connected by edges of a network^1^,^2^,^3^,^4^,^5^,^6^.

One such category is the distance-based descriptors which include Wiener index, Harary index, etc. The use of Wiener index and related type of indices dates back to the seminal work of Wiener in 1947^7^. Wiener introduced his celebrated index to predict the physical properties, such as boiling point, heats of isomerization and differences in heats of vaporization, of isomers of paraffin by their chemical structures. Wiener index has since inspired many distance-based descriptors in Chemometrics. These include Harary index^8^, hyper Wiener index^9^,^10^, Wiener polynomial^11^, Balaban index^12^, Wiener polarity index^7^ and information indices^13^,^14^,^15^. These indices, or commonly called descriptors, play significant roles in quantitative structure-activity relationship/quantitative structure-property relationship (QSAR/QSPR) models. It is known that the Wiener type indices depend both on a network’s number of nodes and its topology. For more results, we refer to^16^,^17^.

Let *G* = (*V*, *E*) be a connected simple graph. The distance between two vertices *u* and *v* in *G*, denoted by *d*_*G*_(*u*, *v*), is the length of a shortest path between *u* and *v* in *G*. The Wiener polarity index of a graph *G* = (*V*, *E*), denoted by *W*_*p*_(*G*), is the number of unordered pairs of vertices {*u*, *v*} of *G* such that *d*_*G*_(*u*, *v*) = 3, i.e.,


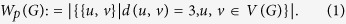


The name “Wiener polarity index” is introduced by Harold Wiener^7^ in 1947. Wiener himself conceived the index only for acyclic molecules and defined it in a slightly different – yet equivalent – manner. In the same paper, Wiener also introduced another index for acyclic molecules, called *Wiener index* or *Wiener distance index* and defined by 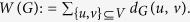 Wiener^7^ used a liner formula of *W* and *W*_*P*_ to calculate the boiling points *t*_*B*_ of the paraffins, *i.e.,* 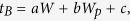 where *a*, *b* and *c* are constants for a given isomeric group. The Wiener index *W*(*G*) is popular in chemical literatures. For more results on Wiener index, we refer to the survey paper^18^ written by Dobrynin, Entringer and Gutman, and some recent papers^19^,^20^,^21^,^22^,^23^.

The Wiener polarity index is used to demonstrate quantitative structure-property relationships in a series of acyclic and cycle-containing hydrocarbons by Lukovits and Linert^24^. Hosoya in^25^ found a physical-chemical interpretation of *W*_*p*_(*G*). Du, Li and Shi^26^ described a linear time algorithm APT for computing the Wiener polarity index of trees, and characterized the trees maximizing the Wiener polarity index among all trees of given order. From then on, the Wiener polarity index started to attract the attention of a remarkably large number of mathematicians and so many results appeared. The extremal Wiener polarity index of (chemical) trees with given different parameters (e.g. order, diameter, maximum degree, the number of pendants, etc.) were studied, see^27^,^28^,^29^,^30^,^31^,^32^,^33^. Moreover, the unicyclic graphs minimizing (resp. maximizing) the Wiener polarity index among all unicyclic graphs of order *n* were given in^34^. There are also extremal results on some other graphs, such as fullerenes, hexagonal systems and cactus graph classes, we refer to^35^,^36^,^37^. Observe that the Wiener polarity index is also related to the cluster coefficient of networks.

## Results

The main contributions of this paper can be summarized as follows:

- We provide a formula of the Wiener polarity index of bicyclic networks, from which the value of the index can be computed easily.
- We introduce three graph transformations, which can be used to increase the values of Wiener polarity index. These transformations can help to find more extremal values for other classes of molecular networks.
- We determine the maximum value of the Wiener polarity index of bicyclic networks and characterize the corresponding extremal graphs.

Now let us introduce some notations. Let *N*_*G*_(*v*) be the neighborhood of *v*, and 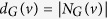 denote the degree of vertex *v*. For 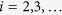, we call 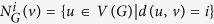 the ith neighborhood of *v*. If *d*_*G*_(*v*) = 1, then we call *v* a *pendant vertex* of *G*. Let *g*(*C*_*x*_) be the length of cycle *C*_*x*_ in graph *G*, *P*_*i*_ denote a path with length *i*. For all other notations and terminology, not given here, see e.g.^38^.

**Figure 1:**
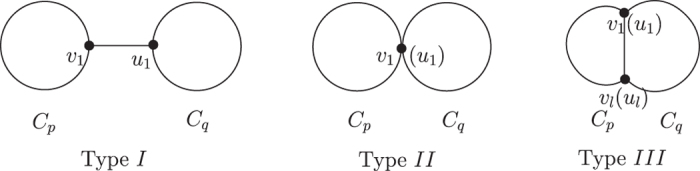
The three types of bicyclic graphs.

Let *B* be a bicyclic graph. Suppose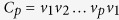 and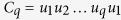 are two cycles in *B* with *l* (*l* ≥ 0) common vertices. Without loss of generality, we label the vertices of *C*_*p*_ in the clockwise direction, and the vertices of *C*_*q*_ in the inverse clockwise direction. If *l* = 0, then there is one unique path *P* connecting *C*_*p*_ and *C*_*q*_, which starts with *v*_1_ and ends with *u*_1_. We call this kind of bicyclic graph **type** ***I*** (see Fig. 1). If *l* = 1, then *C*_*p*_ and *C*_*q*_ have exactly one common vertex *v*_1_(*u*_1_). We call this kind of bicyclic graphs **type** ***II*** (see Fig. 1). If *l*  ≥ 2, then *B* contains exactly three cycles. The third cycle is denoted by *C*_*z*_, where *z* = *p* + *q* − 2*l* + 2. Without loss of generality, assume that *p* ≤ *q* ≤ *z* and *l* − 2 ≤ *p* − 2 ≤ *q* − 2. The two cycles *C*_*p*_ and *C*_*q*_ have more than one common vertex 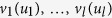. We call this kind of bicyclic graphs **type** ***III*** (see Fig. 1). In the following section, we use *B*, *C*_*p*_, *C*_*q*_, *v*_*i*_ (1 ≤ *i* ≤ *p*), *u*_*j*_ (1 ≤ *j* ≤ *q*), *l* as defined above, except as noted.

Let 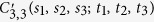 be the bicyclic graph of type *I*, where *P* = *v*_1_*u*_1_ and 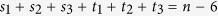. Especially, we denote this kind of graphs by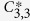, if 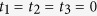, 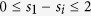 (*i* = 2, 3), 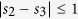. For a graph *G* = (*V*, *E*) and 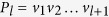, we can construct a new graph *H* by identifying *v*_1_ with 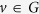, denoted by 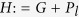, and we say *P*_*l*_ is *incident* to vertex *v*.

**Theorem 0.1.** *Let B*_1_ *be a bicyclic graph in type I and* 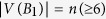, 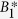 *be the desired graph attaining the maximum Wiener polarity index.*

1. *If n* = 6*, then* 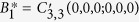
*, and* 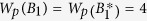;
2. *If n* = 7*, then* 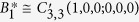
*, and* 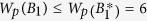;
3. *If n* = 8*, then* 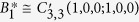,
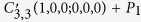
*, where P*_1_ *is incident to the pendant vertex of v*_1_*, and* 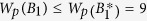;
4. *If n* = 9*, then* 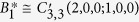,
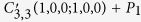
*, where the path P*_1_ *is incident to the pendant vertex of v*_1_,
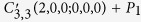
*, where the path P*_1_ *is incident to one pendant vertex of v*_1_, 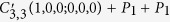
*, where the two paths P*_1_ *are incident to the pendant vertex of v*_1_*, and* 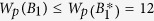;
5. *If n* = 10*, then* 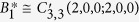,
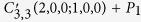
*, where the path P*_1_ *is incident to one pendant vertex of v*_1_, 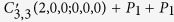
*, where the two paths P*_1_ *are incident to the pendant vertices of v*_1_*, and* 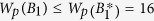;
6. *If n* = 11*, then* 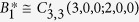,
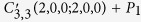
*, where the path P*_1_ *is incident to one pendant vertex of v*_1_,
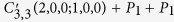
*, where the two paths P*_1_ *are incident to the pendant vertices of v*_1_, 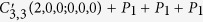
*, where the three paths P*_1_ *are incident to the pendant vertices of v*_1_*, and* 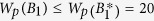;
7. *If n* = 12*, then* 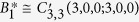, 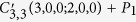
*, where P*_1_ *is incident to one pendant vertex of v*_1_, 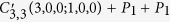
*, where the two paths P*_1_ *are incident to the pendant vertices of v*_1_, 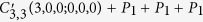
*, where the three paths P*_1_ *are incident to the pendant vertices of v*_1_*, and* 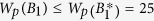;
8. *If n* = 13*, then* 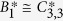, 
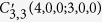
, 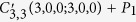
*, where P*_1_ *is incident to one pendent vertex of v*_1_, 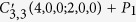
*, where P*_1_ *is incident to one pendant vertex of v*_1_,
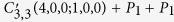
*, where the two paths P*_1_ *are incident to the pendant vertices of v*_1_, 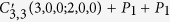
*, where the two paths P*_1_ *are incident to the pendant vertices of v*_1_, 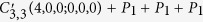
*, where the three paths P*_1_ *are incident to the pendant vertices of v*_1_,
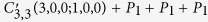
*, where the three paths P*_1_ *are incident to the pendant vertices of v*_1_,
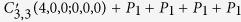
*, where the four paths P*_1_ *are incident to the pendant vertices of v*_1_*, and* 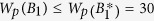;
9. *If n* = 14*, then* 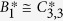, 
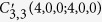
, 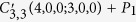
*, where P*_1_ *is incident to one pendent vertex of v*_1_, 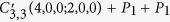
*, where the two paths P*_1_ *are incident to the pendant vertices of v*_1_, 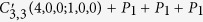
*, where the three paths P*_1_ *are incident to the pendant vertices of v*_1_, 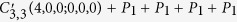
*, where the four paths P*_1_ *are incident to the pendant vertices of v*_1_*, and* 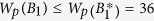;
10. *If n* ≥ 15*, then* 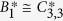
*, and* 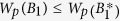. □

Let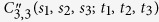 be the bicyclic graph in type *II*, where 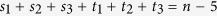, and *s*_1_ = *t*_1_. When *n* is large enough, it can be easily checked that the graph maximizing the Wiener polarity index is 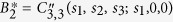 (see support information).

**Theorem 0.2.** *Let B*_2_ *be a bicyclic graph in type II and* 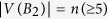, 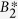 *be the desired graph attaining the maximum Wiener polarity index.*

1. *If n* = 5*, then* 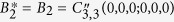
*, and W*_*p*_(*B*_2_) = 0;
2. *If n* = 6*, then* 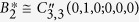,
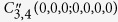
*, and* 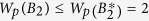;
3. *If n* = 7*, then* 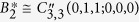,
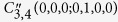
*, and* 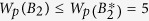;
4. *If n* = 8*, then* 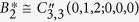,
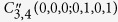
*, and* 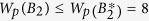;
5. *For n* ≥ 9*, let* 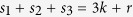 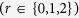.

*If r* = 0*, then* 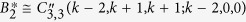,
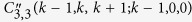
*, and* 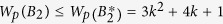;

*If r* = 1*, then* 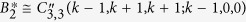
*, and* 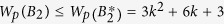;

*If r* = 2*, then* 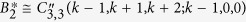
*, and* 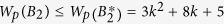.□

Let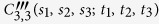 be the bicyclic graph in type *III*, where 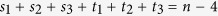, *s*_1_ = *t*_1_, *s*_2_ = *t*_1_ and *l* = 1. Let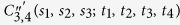 be the bicyclic graph in type *III*, where 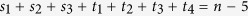, *s*_1_ = *t*_1_, *s*_2_ = *t*_1_ and *l* = 1. When *n* is large enough, it can be checked that the graph maximizing the Wiener polarity index is 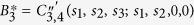.

**Theorem 0.3.** *Let B*_3_ *be a bicyclic graph in type III and*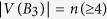, 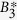 *be the desired graph attaining the maximum Wiener polarity index.*

1. *If n* = 4*, then* 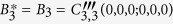
*, and W*_*p*_(*B*_3_) = 0;
2. *If n* = 5*, then* 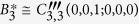
*, and* 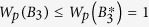;
3. *If n* = 6*, then* 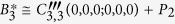
*, where P*_2_ *is incident to vertex v*_1_ *or v*_3_*, and* 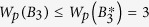;
4. *If n* = 7*, then* 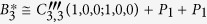
*, where the two paths P*_1_ *are incident to the pendant vertex of v*_1_*, and* 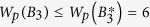;
5. *If n* = 8*, then* 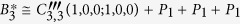
*, where the three paths P*_1_ *are incident to the pendant vertex of v*_1_*, and* 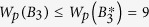;
6. *If n* = 9*, then* 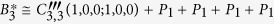
*, where the four paths P*_1_ *are incident to the pendant vertex of v*_1_, 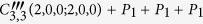
*, where the three paths P*_1_ *are incident to the pendant vertices of v*_1_*, and* 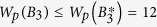;
7. *If n* = 10*, then* 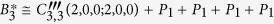
*, where the four paths P*_1_ *are incident to the pendant vertices of v*_1_*, and* 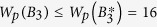;
8. *If n* = 11*, then* 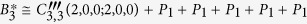
*, where the five paths P*_1_ *are incident to the pendant vertices of v*_1_,
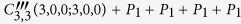
*, where the four paths P*_1_ *are incident to the pendant vertices of v*_1_,
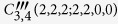
,
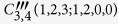
*, and* 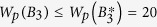;
9. *For n* ≥ 12*, let* 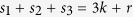 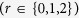.

*If r* = 0*, then* 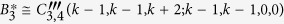, 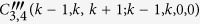
*, and* 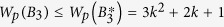;

*If r* = 1*, then* 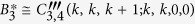,
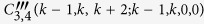
*, and* 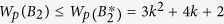;

*If r* = 2*, then* 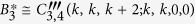
*, and* 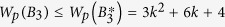. □

**Theorem 0.4.** *Let B be a bicyclic graph of order n* (≥4), B^*^ *be the bicyclic graph with the maximum polarity index among all bicyclic graphs.*

1. *If n* = 4*, then* 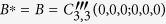
*, and W*_*p*_(*B*_3_) = 0;
2. *If n* = 5*, then* 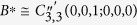
*, and* 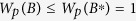;
3. *If n* = 6*, then* 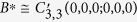
*, and* 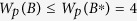;
4. *If n* = 7*, then* 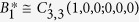, 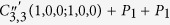
*, where the two paths P*_1_ *are incident to the pendant vertex of v*_1_ *and* 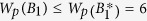;;
5. *If n* = 8*, then* 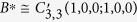, 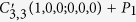
*, where P*_1_ *is incident to one pendant vertex of v*_1_, 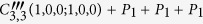
*, where the three paths P*_1_ *are incident to the pendant vertex of v*_1_*, and* 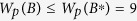;
6. *If n* = 9*, then* 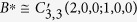, 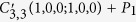
*, where the path P*_1_ *is incident to the pendant vertex of v*_1_, 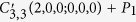
*, where the path P*_1_ *is incident to one pendant vertex of v*_1_, 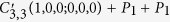
*, where the two paths P*_1_ *are incident to the pendant vertex of v*_1_, 
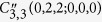
, 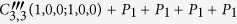
*, where the four paths P*_1_ *are incident to the pendant vertex of v*_1_, 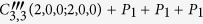
*, where the three paths P*_1_ *are incident to the pendant vertices of v*_1_*, and* 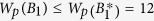;
7. *If n* = 10*, then* 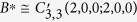, 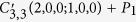
*, where the path P*_1_ *is incident to one pendant vertex of v*_1_, 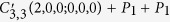
*, where the two paths P*_1_ *are incident to the pendant vertices of v*_1_, 
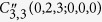
, 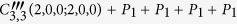
*, where the four paths P*_1_ *are incident to the pendant vertices of v*_1_*, and* 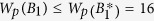;
8. *For n* ≥ 11*, let* 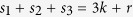 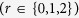.

*If r* = 0*, then* 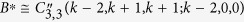, 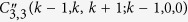
*, and* 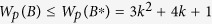;

*If r* = 1*, then* 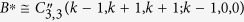
*, and* 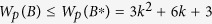;

*If r* = 2*, then* 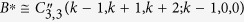
*, and* 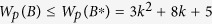. □

## Discussion

Quantifying the structure of complex networks is still intricate because the structural interpretation of quantitative network measures and their interrelations have not yet been explored extensively. In this paper, we studied sharp upper bounds for the Wiener polarity index among all bicyclic networks, by using some transformations. The graphs attaining these bounds are also characterized. The proof techniques use structural properties of the graphs under consideration and it may be intricate to extend the techniques when using more general graphs.

An interesting thing is that the Wiener polarity index is related to a pure mathematical problem: counting the number of subgraphs of a graph. This counting problem is a basic problem in mathematics but much more complicated. For example, Alon and Bollobás provide some results on this topic, e.g.^39^,^40^,^41^.

As a future work, we will consider the extremal problems of the Wiener polarity index for general networks and also some special networks. Furthermore, we would like to explore advanced structural properties of the Wiener polarity index, and relations between the Wiener polarity index and some other topological indices. On the other hand, it would be interesting to investigate the applications of Wiener polarity index in characterizing the structure properties of complex networks and studying algorithm theory and computational complexity. For instance, one can consider the possibility of using the Wiener polarity index or other distance measures to study other very interesting algorithms, like the google algorithm in complex networks^42^,^43^.

## Methods

First we introduce some operations on bicyclic graphs, then we give the corresponding lemmas which state that the Wiener polarity index is not decreasing after applying these operations on bicyclic graphs.

Let *B* be a bicyclic graph. As we have claimed, suppose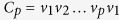 and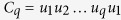 are two cycles. If both 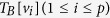 and 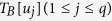 are stars, then we denote such a bicyclic graph by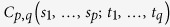, where *s*_*i*_ and *t*_*j*_ represent the number of pendant vertices of *v*_*i*_ and *u*_*j*_, respectively.

We define **Operation** ***I*** as follows. Let *T*_*B*_[*v*] denote a hanging tree on vertex *v* of a bicyclic graph *B* with *p* ≥ 4, *q* ≥ 4, where *v* is on the cycle of *B*. Among all hanging trees, suppose 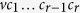 is one of the longest paths from the root *v* to a leaf *c*_*r*_ in *T*_*B*_[*v*]. If *r* ≥ 2, then after deleting the edge *vc*_1_ from *B*, we obtain a bicyclic graph *A* and a tree *T* such that 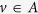 and 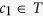. Let B^*^ denote the bicyclic graph obtained from *A* and *T* by identifying *c*_1_ and *v*′ (a neighbor of *v* on the cycle of *B*) and adding a new hanging leaf *vx* to *v*.

We define **Operation** ***II*** as follows. Let *B* be a bicyclic graph with *p* = 3, *T*_*B*_[*v*_*i*_] be a hanging tree rooted at *v*_*i*_ (*i* = 1, 2, 3). Let 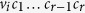 be one of the longest paths from the root *v*_*i*_ to a leaf *c*_*r*_ of the hanging tree *T*_*B*_[*v*_*i*_].

For *r* ≥ 3, we define a new graph B^*^ as follows:


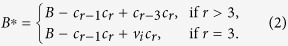


For *r* = 2, the operation differs on the three types of bicyclic graphs.

(1) For bicyclic graphs in type *I*, we let


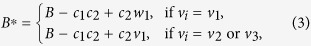


where 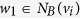 is on the path 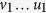.

(2) For bicyclic graphs in type *II*, by considering the value of *q*, there are two cases.

**Case 1.** *q* ≥ 4. In this case, let


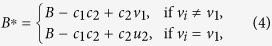


where 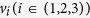 is the root vertex mentioned above.

**Case 2.** *q* = 3 and 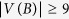. Here we let *C*_*q*_ = *v*_1_*v*_4_*v*_5_*v*_1_. We define an operation as follows: delete *T*_*B*_[*v*_*i*_]\*v*_*i*_ and add a copy of *T*_*B*_[*v*_*i*_] to *v*_*j*_ by identifying *v*_*j*_ and 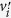 which is a copy of *v*_*i*_. We call this operation “move *T*_*B*_[*v*_*i*_] to *v*_*j*_”. By considering the number of vertices on the cycles of *B* with hanging trees, there are two subcases.

**Subcase 2.1.** There is only one vertex 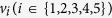 with a hanging tree. Let 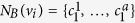, 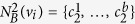.

For the case *v*_*i*_ = *v*_1_, we apply operations as follows. If *b* ≥ 4, then move 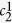, 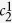 to *v*_2_ and 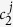 (3 ≤ *j* ≤ *b*) to *v*_3_; if *b* = 3, then move 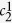, 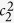 to *v*_2_ and 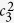, 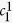 to *v*_3_; if *b* = 2, then move 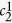, 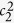 to *v*_2_ and 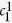 to *v*_3_; if *b* = 1, then move 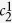 to *v*_2_ and 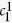 to *v*_3_. The new graph is denoted by B^*^.

For the case *v*_*i*_ = *v*_2_, we construct a new graph 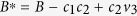.

**Subcase 2.2.** There are at least two vertices *v*_*s*_, *v*_*t*_ 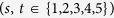 with hanging trees. In this subcase, let 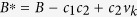, where 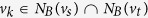.

(3) For the bicyclic graphs in type *III*. By considering the value of *q*, there are two cases.

**Case 1.** *q* ≥ 4. In this case, we can apply Operation 1 on *C*_*z*_.

**Case 2.** *q* = 3 and 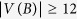. Here let *C*_*q*_ = *v*_1_*v*_2_*v*_4_*v*_1_. We can move *T*_*B*_[*v*_4_] to *v*_3_ to get a new graph *B*′ satisfying *W*_*p*_(*B*′) = *W*_*p*_(*B*). By considering the number of vertices on the cycles of *B*′ with hanging trees, there are two subcases.

**Subcase 2.1.** There exists only one vertex, say 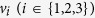, which has a hanging tree. Firstly, move *T*_*B*′_[*v*_*i*_] to *v*_3_ (denote the new graph by *B*″), delete a vertex in 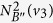 and meanwhile subdivide edge *v*_1_*v*_4_ (denote the new graph by 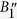); secondly, move all the other vertices in 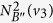 to *v*_1_ (denote the new graph by *B*″′); thirdly, if 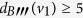, then just move one pendant vertex of *v*_1_ to *v*_2_; if 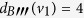, then move one pendant vertex of *v*_3_ to *v*_2_.

**Subcase 2.2.** There exist two vertices, say 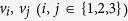, which have hanging trees. If *i* = 1 and *j* = 2, then move *T*_*B*′_[*v*_2_] to *v*_3_. Now we can only consider the case *i* = 1 and *j* = 3.

If there exists 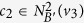, then delete *c*_2_ and subdivide the edge *v*_1_*v*_4_ (denote the new graph by *B*″). Now return to the situation in Case 1.

If and 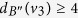, then delete a vertex 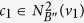 and subdivide the edge *v*_1_*v*_4_. Now return to Case 1. For the situation that 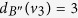, delete a vertex 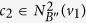 and subdivide the edge *v*_1_*v*_4_, move all pendant vertices in 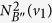 to *v*_2_, at last move one pendant vertex of *v*_1_ or *v*_2_ to *v*_3_.

**Subcase 2.3.** There exist three vertices which have hanging trees. By deleting some pendant vertex in 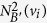, where 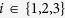, and meanwhile subdividing the edge *v*_1_*v*_4_, we return to the situation in Case 1.

The final graph obtained after the above operation is denoted by B^*^.

We define **Operation** ***III*** as follows. Let *B* be a bicyclic graph. If *d*_*B*_(*v*) = 2, then let 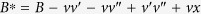, where *v*′, 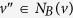, 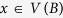. We call such an operation *smooth v* to *x*.

We define **Operation** ***IV*** as follows. Let *B* be a bicyclic graph, where 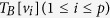 and 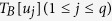 are both stars. Denote the set of the pendant vertices of *v*_*i*_(*u*_*j*_) by *V*_*i*_(*U*_*j*_).

For bicyclic graphs in type *I*, we will take the following two steps.

**Step 1.** For *C*_*p*_ and 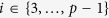, if *i* is odd, then move *V*_*i*_ to *v*_1_ and smooth *v*_*i*_ to *v*_2_; if *i* is even, then move *V*_*i*_ to *v*_2_ and smooth *v*_*i*_ to *v*_1_. For *C*_*q*_ and 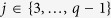, if *j* is odd, then move *U*_*j*_ to *u*_1_ and smooth *u*_*j*_ to *u*_2_; if *j* is even, then move *U*_*j*_ to *u*_2_ and smooth *u*_*j*_ to *u*_1_. Therefore, we obtain a graph 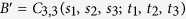 with a unique path *P* connecting *C*_*p*_ and *C*_*q*_. Let the set of hanging leaves of *u*_1_, *u*_2_, *u*_*q*_ be 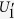, 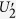, 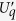, respectively.

**Step 2.** Let 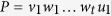, 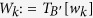 (1 ≤ *k* ≤ *t*).

If *k* is odd, then move *W*_*k*_ to *v*_3_ and smooth *w*_*k*_ to *v*_2_; if *k* is even, then move *W*_*k*_ to *v*_2_ and smooth *w*_*k*_ to *v*_3_.

If *t* is odd, then move 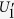 to *v*_2_, 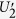 to *v*_1_, 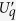 to *v*_3_; if *t* is even and *t* ≥ 2, then move 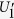 to *v*_3_, 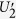 to *v*_1_, and 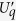 to *v*_2_, respectively; if *t* = 0, 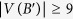 and *d*(*v*_2_) = *d*(*v*_3_) = 2, let 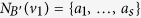 and 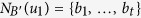, then for the situation that *b* = 1, move *b*_1_ to *v*_2_ and move *a*_1_ to *v*_3_, for the situation that *b* ≥ 2, move *b*_1_ to *v*_2_ and move 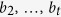 to *v*_3_; if *t* = 0 and *d*(*v*_*i*_) = 2, 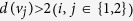, then move 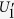 to *v*_*i*_, 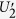 to *v*_1_, 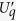 to *v*_*j*_, respectively.

Finally, we get a new graph 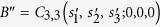 and there is a unique path *P* = *v*_1_*u*_1_ connecting *C*_*p*_ and *C*_*q*_.

For bicyclic graphs in type *II*, we also give two steps as follows.

**Step 1.** For *C*_*p*_ and 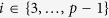, if *i* is odd, then move *V*_*i*_ to *v*_1_ and smooth *v*_*i*_ to *v*_2_; if *i* is even, then move *V*_*i*_ to *v*_2_ and smooth *v*_*i*_ to *v*_1_. For *C*_*q*_ and 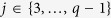, if *j* is odd, then move *U*_*j*_ to *u*_1_ and smooth *u*_*j*_ to *u*_2_; if *j* is even, then move *U*_*j*_ to *u*_2_ and smooth *u*_*j*_ to *u*_1_. Thus we get a graph 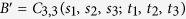 with *s*_1_ = *t*_1_. Let the set of hanging leaves of *u*_1_, *u*_2_, *u*_*q*_ be 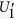, 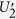, 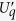, respectively.

**Step 2.** By moving 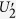 to *v*_2_, 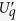 to *v*_*p*_, we have 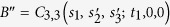 with *s*_1_ = *t*_1_.

For bicyclic graphs in type *III*, the operation is defined as follows. Recall that we use *l* (≥1) to denote the number of common vertices of *C*_*p*_ and *C*_*q*_, and without loss of generality, assume *l* − 2 ≤ *p* − 2 ≤ *q* − 2.

1. If *p* ≥ 3 and *q* ≥ 4, then we will take the following three steps. **Step 1.** For 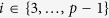, 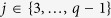. If *i* is odd, then move *V*_*i*_ to *v*_1_; if *i* is even, then move *V*_*i*_ to *v*_2_; if *j* is odd, then move *U*_*j*_ to *v*_1_; if *j* is even, then move *U*_*j*_ to *v*_2_; move *U*_*q*_ to *v*_*p*_. **Step 2.** If *l* = 2 or 3, smooth vertices 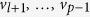 to *v*_1_ and *v*_2_ alternately. If *l* ≥ 4, then we first smooth vertices 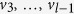 to *v*_1_ and *v*_2_ alternately; then smooth vertices 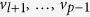 to *v*_1_ and *v*_2_ alternately. After applying this operation, we get a new graph *B*′ with cycles *C*_*p*′_, *C*_*q*′_ and *C*_*z*′_. Let *l*′ be the number of common vertices of *C*_*p*′_ and *C*_*q*′_, *p*′ (*p*′ = 3 *or* 4) be the number of vertices of the smallest cycle of *B*′, then we have *l*′ = 2 or *l*′ = 3. Now relabel the vertices on *C*_*p*′_ and *C*_*q*′_ of *B*′, and we have 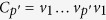 and 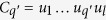. **Step 3.** Considering the value of *l*′, there are two cases. **Case 1.** *l*′ = 2. We just smooth 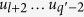 to *v*_1_ and *v*_2_ alternately, and smooth *u*_*q*′−1_ to *v*_*p*′_. The new graph obtained is denoted by 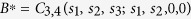. **Case 2.** *l*′ = 3, 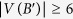 and 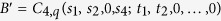 with *s*_1_ = *t*_1_. Let 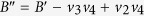. If *q*′ ≥ 5, then smooth *v*_3_ to vertex *v*_1_, smooth 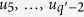 to *v*_1_ and *v*_2_ alternately and smooth *u*_*q*′−1_ to *v*_4_; if *q*′ = 4 and 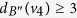, then we do nothing; if *q*′ = 4 and 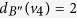, then move the pendant vertices of *v*_2_ to *v*_4_. Finally, we get the desired graph 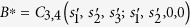.
2. If *p* = 3 and *q* = 3, by Operation *II* on *B* and its resultant graphs repeatedly, we have a new graph 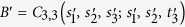. Move the pendant vertices of *u*_3_ to *v*_3_, we obtain 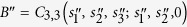.

## Additional Information

**How to cite this article**: Ma, J. *et al.* On Wiener polarity index of bicyclic networks. *Sci. Rep.* **6**, 19066; doi: 10.1038/srep19066 (2016).

## Supplementary Material

Supplementary Information
