# Supplementary material for: On Wiener polarity index of bicyclic networks
Source: Sci Rep. 2016 Jan 11;6:19066. doi: 10.1038/srep19066 (PMC4707490; doi:10.1038/srep19066)
Supplement: Supplementary Information [file srep19066-s1.pdf]

On Wiener polarity index of bicyclic networks Supplementary Information

Jing Ma[1], **Yongtang Shi**[1,*], **Zhen Wang**[2], **and Jun Yue**[3]

[1]Center for Combinatorics and LPMC-TJKLC, Nankai University, Tianjin, 300071, China
[2]Interdisciplinary Graduate School of Engineering Sciences, Kyushu University, Kasuga-koen, Kasugashi, Fukuoka 816-8580, Japan
[3]School of Mathematical Sciences, Shandong Normal University, Jinan 250014, Shandong, China
[*]shi@nankai.edu.cn

ABSTRACT

1Proofs for the theorems

Now let us introduce some notations. Let $N_G(v)$ be the neighborhood of v, and $d_G(v) = |N_G(v)|$ denote the degree of vertex v. For $i = 2,3,\ldots$, we call $N_G^i(v) = \{u \in V(G) | d(u,v) = i\}$ the ith neighborhood of v. If $d_G(v) = 1$, then we call v a *pendant vertex* of G. Let $g(C_x)$ be the length of cycle C_x in graph G, P_i denote a path with length i. For all other notations and terminology, not given here, see e.g.[1]

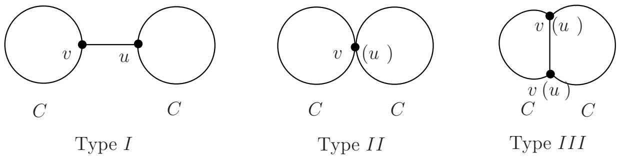

Figure 1. The three types of bicyclic graphs.

Let B be a bicyclic graph. Suppose $C_p = v_1v_2\ldots v_pv_1$ and $C_q = u_1u_2\ldots u_qu_1$ are two cycles in B with l $(l \geq 0)$ common vertices. Without loss of generality, we label the vertices of C_p in the clockwise direction, and the vertices of C_q in the inverse clockwise direction. If $l = 0$, then there is one unique path P connecting C_p and C_q, which starts with v_1 and ends with u_1. We call this kind of bicyclic graph **type** I (see Figure 1). If $l = 1$, then C_p and C_q have exactly one common vertex v_1 (u_1). We call this kind of bicyclic graphs **type** II (see Figure 1). If $l \geq 2$, then B contains exactly three cycles. The third cycle is denoted by C_z, where $z = p+q-2l+2$. Without loss of generality, assume that $p \leq q \leq z$ and $l-2 \leq p-2 \leq q-2$. The two cycles C_p and C_q have more than one common vertex $v_1(u_1),\ldots,v_l(u_l)$. We call this kind of bicyclic graphs **type** III (see Figure 1). In the following section, we use B, C_p, C_q, v_i $(1 \leq i \leq p)$, u_j $(1 \leq j \leq q)$, l as defined above, except as noted.

Now we first recall two useful lemmas on the Wiener polarity index of trees and unicyclic graphs.

Lemma 0.1. [2] *Let $T = (V,E)$ be a tree. Then $W_p(T) = \sum_{uv \in E} (d_T(u)-1)(d_T(v)-1)$.* □

Lemma 0.2. [3] *Let $U = (V,E)$ be a unicyclic graph. Let C denote the unique cycle of U. If $g(U) = 3$ with $V(C) = \{v_1,v_2,v_3\}$, then*

$$W_p(U) = \sum_{uv \in E} (d_U(u)-1)(d_U(v)-1) + 9 - 2d_U(v_1) - 2d_U(v_2) - 2d_U(v_3). \tag{1}$$

If $g(U) = 4$ and $V(C) = \{v_1,v_2,v_3,v_4\}$, then

$$W_p(U) = \sum_{uv \in E} (d_U(u)-1)(d_U(v)-1) + 4 - d_U(v_1) - d_U(v_2) - d_U(v_3) - d_U(v_4). \tag{2}$$

Moreover, if $g(U) \geq 5$, *then*

$$W_p(U) = \begin{cases} \sum\limits_{uv\in E}(d_U(u)-1)(d_U(v)-1)-5, & \textit{if } g(U)=5; \\ \sum\limits_{uv\in E}(d_U(u)-1)(d_U(v)-1)-3, & \textit{if } g(U)=6; \\ \sum\limits_{uv\in E}(d_U(u)-1)(d_U(v)-1), & \textit{if } g(U)\geq 7. \end{cases} \quad (3)$$

□

In the following, we introduce an operation for the three types of bicyclic graphs. We call this operation *graph decomposition*. A *hanging tree* on vertex v in B, denoted by $T_B[v]$, is a rooted tree whose root is the vertex v. For a bicyclic graph B of type I, $T_B[v_1]$ ($T_B[u_1]$) is a rooted tree without path P. Especially, when $T_B[v]$ is a star, we call the edges incident to the vertex v *hanging leaves* of v.

Firstly, we consider bicyclic graphs of type I. Let $N_B(v_1) = \{a_1,\ldots,a_s,w,v_2,v_p\}$, where w is on the unique path P connecting C_p and C_q. Now we decompose B into two unicyclic graphs U_1 and U_2 as follows (see Figure 2): separate vertex $v_1 \in V(B)$ into two vertices $v_1^0 \in V(U_1)$ and $v_1' \in V(U_2)$; add vertex w' (a copy of w) to U_1 and join w' to v_1^0; add vertices $v_2', v_p', a_1', \ldots, a_s'$ (copies of $v_2, v_p, a_1, \ldots, a_s$) to U_2 and join $v_2', v_p', a_1', \ldots, a_s'$ to v_1'. Then we have $T_{U_1}[v_1^0] = T_B[v_1] \cup \{v_1^0 w'\}$. Observe that $T_{U_2}[v_1']$ is a star.

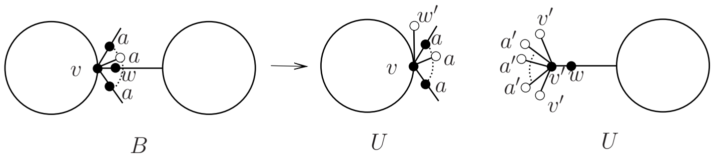

Figure 2. The decomposition of type I.

Now we consider bicyclic graphs of type II. Let $N_B(v_1) = \{a_1,\ldots,a_s,v_2,v_p,u_2,u_q\}$. We decompose B into two unicyclic graphs U_1 and U_2 as follows (see Figure 3): separate the common vertex $v_1 \in V(B)$ into two vertices $v_1^0 \in V(U_1)$ and $u_1^0 \in V(U_2)$; add vertices u_2', u_q' (copies of u_2, u_q) to U_1 and join u_2', u_q' to v_1^0; add vertices $v_2', v_p', a_1', \ldots, a_s'$ (copies of $v_2, v_p, a_1, \ldots, a_s$) to U_2, and join $v_2', v_p', a_1', \ldots, a_s'$ to u_1^0. Then we have $T_{U_1}[v_1^0] = T_B[v_1] \cup \{v_1^0 u_2', v_1^0 u_q'\}$. Observe that $T_{U_2}[u_1^0]$ is a star.

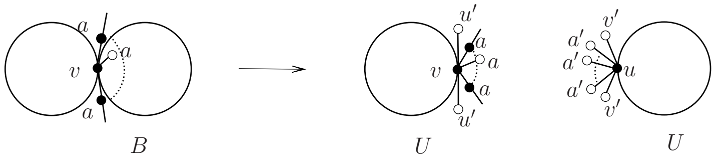

Figure 3. The decomposition of type II.

Finally, we consider bicyclic graphs of type III. Let $N_B(v_1) = \{a_1,\ldots,a_s,v_2(u_2),v_p,u_q\}$, $N_B(v_l) = \{b_1,\ldots,b_t,v_{l-1}(u_{l-1}), v_{l+1},u_{l+1}\}$. Now we decompose B into two unicyclic graphs U_1 and U_2 as follows (see Figure 4): separate vertex $v_i \in V(B)$ into two vertices $v_i^0 \in V(U_1)$ and $u_i^0 \in V(U_2)$ $(1 \leq i \leq l)$; add vertex u_q' (a copy of u_q) to U_1 and join u_q' to v_1^0; add vertex u_{l+1}' (a copy of u_{l+1}) to U_1, and join u_{l+1}' to v_l^0; add vertices $v_p', a_1', \ldots, a_s'$ (copies of $v_p, a_1, \ldots, a_s$) to U_2, and join $v_p', a_1', \ldots, a_s'$ to u_1^0; add vertices $v_{l+1}', b_1', \ldots, b_t'$ (copies of $v_{l+1}, b_1, \ldots, b_t$) to U_2, and join $v_{l+1}', b_1', \ldots, b_t'$ to u_l^0; join u_k^0 to u_{k+1}^0 $(1 \leq k \leq l-1)$ in U_2. Then we have $T_{U_1}[v_1^0] = T_B[v_1] \cup \{v_1^0 u_q'\}$, $T_{U_1}[v_l^0] = T_B[v_l] \cup \{v_l^0 u_{l+1}'\}$, $T_{U_1}[v_i^0] = T_B[v_i]$ $(2 \leq i \leq l-1)$, $d_{U_2}(u_j^0) = 2$ $(2 \leq j \leq l-1)$. Observe that $T_{U_2}[u_1^0]$ and $T_{U_2}[u_l^0]$ are stars.

Lemma 0.3. *Let B be a bicyclic graph, and U_1, U_2 be the two unicyclic graphs obtained from B by the graph decomposition mentioned above.*

(1) If $l = 0$, then $W_p(B) = W_p(U_1) + W_p(U_2)$;

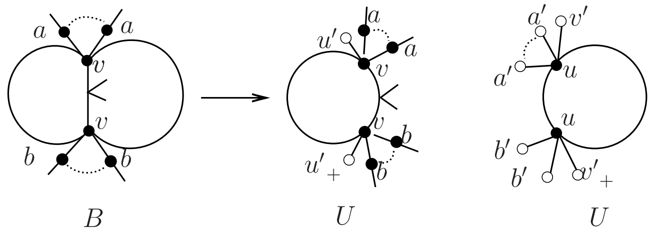

Figure 4. The decomposition of type III.

(2) If $l=1$, then $W_p(B)=W_p(U_1)+W_p(U_2)$;
(3) If $l\geq 2$ and $z=p+q-2l+2\geq 7$, then

$$W_p(B)=W_p(U_1)+W_p(U_2)-\sum_{\substack{1\leq i\leq l-1\\ u_i^0u_{i+1}^0\in U_2}}(d_{U_2}(u_i^0)-1)(d_{U_2}(u_{i+1}^0)-1). \tag{4}$$

Proof. (1) For a graph G, define $D_e(G)=\{\{u,v\}|d_G(u,v)=3\}$ and there is a shortest path from u to v passing through the edge $e=ab$, where $a,b,u,v\in V(G)\}$. Then for a unicyclic graph $U=(V,E)$, we have

$$W_p(U)=\begin{cases}\sum_{e\in E}|D_e(U)|+9-2d_U(v_1)-2d_U(v_2)-2d_U(v_3), & \text{if } g(U)=3,\\ \sum_{e\in E}|D_e(U)|+4-d_U(v_1)-d_U(v_2)-d_U(v_3)-d_U(v_4), & \text{if } g(U)=4,\\ \sum_{e\in E}|D_e(U)|-5, & \text{if } g(U)=5,\\ \sum_{e\in E}|D_e(U)|-3, & \text{if } g(U)=6,\\ \sum_{e\in E}|D_e(U)|, & \text{if } g(U)\geq 7.\end{cases} \tag{5}$$

Suppose $N_B(v_1)=\{a_1,\dots,a_s,x,v_2,v_p\}$. Using the graph decomposition for type I, we obtain that $|D_{v_1v_2}(B)|=|D_{v_1^0v_2}(U_1)|$, $|D_{v_1v_p}(B)|=|D_{v_1^0v_p}(U_1)|$, $|D_{v_1w}(B)|=|D_{v_1'w}(U_2)|$, $|D_{v_1^0w'}(U_1)|=0$, $|D_{v_1'v_2'}(U_2)|=0$, $|D_{v_1'v_p'}(U_2)|=0$, $|D_{v_1a_i}(B)|=|D_{v_1^0a_i}(U_1)|$ $(1\leq i\leq s)$, $|D_{v_1'a_i^0}(U_2)|=0$ $(1\leq i\leq s)$. For the other edges uv in B, the corresponding edges are either in U_1 or U_2, and we can directly check that either $|D_{uv}(B)|=|D_{uv}(U_1)|$ or $|D_{uv}(B)|=|D_{uv}(U_2)|$. Therefore, $W_p(B)=W_p(U_1)+W_p(U_2)$.

The proof of (2) and (3) are just similar to the proof of (1). □

By Lemma 0.3, we can obtain the formula of the Wiener polarity index of bicyclic graphs. Let B be a bicyclic graph with two cycles $C_p=v_1v_2\dots v_pv_1$ and $C_q=u_1u_2\dots u_qu_1$. Suppose U_1 and U_2 are the corresponding unicyclic graphs obtained from graph decomposition. Let

$$x_i=\begin{cases}9-2d_B(v_1)-2d_B(v_2)-2d_B(v_3), & \text{if } i=3,\\ 4-d_B(v_1)-d_B(v_2)-d_B(v_3)-d_B(v_4), & \text{if } i=4,\\ -5, & \text{if } i=5,\\ -3, & \text{if } i=6,\\ 0, & \text{if } i\geq 7,\end{cases} \tag{6}$$

and

$$y_j=\begin{cases}9-2d_B(u_1)-2d_B(u_2)-2d_B(u_3), & \text{if } j=3,\\ 4-d_B(u_1)-d_B(u_2)-d_B(u_3)-d_B(u_4), & \text{if } j=4,\\ -5, & \text{if } j=5,\\ -3, & \text{if } j=6,\\ 0, & \text{if } j\geq 7.\end{cases} \tag{7}$$

Then for type I, we have

$$W_p(B) = W_p(U_1) + W_p(U_2) = \sum_{uv \in E(B)} (d_B(u)-1)(d_B(v)-1) + x_i + y_j. \tag{8}$$

For type II, we have

$$W_p(B) = W_p(U_1) + W_p(U_2) = \sum_{uv \in E(B)} (d_B(u)-1)(d_B(v)-1) + x_i + y_j, \tag{9}$$

where $v_1 = u_1$. For type III, if $z \geq 7$, we have

$$W_p(B) = \sum_{uv \in E(B)} (d_B(u)-1)(d_B(v)-1) + x_i + y_j, \tag{10}$$

where $1 \leq m \leq l-1$ and $v_k = u_k$ $(1 \leq k \leq l)$. If $z < 7$, we have

$$W_p(B) = \begin{cases} \sum\limits_{uv}(d_B(u)-1)(d_B(v)-1) - 4d_B(v_1) - 4d_B(v_2) - 3d_B(v_3) - 3d_B(u_3) + 24, & \text{for } (3,3,2); \\ \sum\limits_{uv}(d_B(u)-1)(d_B(v)-1) - 3d_B(v_1) - 3d_B(v_2) - 2d_B(v_3) - d_B(u_3) - d_B(u_4) + 13, & \text{for } (3,4,2); \\ \sum\limits_{uv}(d_B(u)-1)(d_B(v)-1) - 2d_B(v_1) - 2d_B(v_2) - 2d_B(v_3) + 3, & \text{for } (3,5,2); \\ \sum\limits_{uv}(d_B(u)-1)(d_B(v)-1) - 2d_B(v_1) - 2d_B(v_2) - d_B(v_3) - d_B(v_4) - d_B(u_3) - d_B(u_4) + 24, & \text{for } (4,4,2); \\ \sum\limits_{uv}(d_B(u)-1)(d_B(v)-1) - 2d_B(v_1) - 2d_B(v_2) - 2d_B(v_3) - 2d_B(v_4) - 2d_B(u_4) + 12, & \text{for } (4,4,3); \\ \sum\limits_{uv}(d_B(u)-1)(d_B(v)-1) - d_B(v_1) - d_B(v_2) - d_B(v_3) - d_B(v_4) - 3, & \text{for } (4,5,3); \\ \sum\limits_{uv}(d_B(u)-1)(d_B(v)-1) - d_B(v_1) - d_B(v_2) - d_B(v_3) - d_B(v_4), & \text{for } (4,6,3); \\ \sum\limits_{uv}(d_B(u)-1)(d_B(v)-1) - 12, & \text{for } (5,5,3), \end{cases} \tag{11}$$

where $uv \in E(B)$ and (a,b,c) denotes the values of p, q and l, respectively.

1Methods and Results

In this section, we characterize the bicyclic graphs of given order maximizing the index among all bicyclic graphs. First we introduce some operations on bicyclic graphs, then we give the corresponding lemmas which state that the Wiener polarity index is not decreasing after applying these operations on bicyclic graphs.

Let B be a bicyclic graph. As we have claimed, suppose $C_p = v_1v_2\ldots v_pv_1$ and $C_q = u_1u_2\ldots u_qu_1$ are two small cycles. If both $T_B[v_i]$ $(1 \leq i \leq p)$ and $T_B[u_j]$ $(1 \leq j \leq q)$ are stars, then we denote such a bicyclic graph by $C_{p,\,q}(s_1,\ldots,s_p;t_1,\ldots,t_q)$, where s_i and t_j represent the number of pendant vertices of v_i and u_j, respectively.

We define **Operation** I (see Figure 5) as follows. Let $T_B[v]$ denote a hanging tree on vertex v of a bicyclic graph B with $p \geq 4$, $q \geq 4$, where v is on the cycle of B. Among all hanging trees, suppose $vc_1\ldots c_{r-1}c_r$ is one of the longest paths from the root v to a leaf c_r in $T_B[v]$. If $r \geq 2$, then after deleting the edge vc_1 from B, we obtain a bicyclic graph A and a tree T such that $v \in A$ and $c_1 \in T$. Let B^* denote the bicyclic graph obtained from A and T by identifying c_1 and v' (a neighbor of v on the cycle of B) and adding a new hanging leaf vx to v.

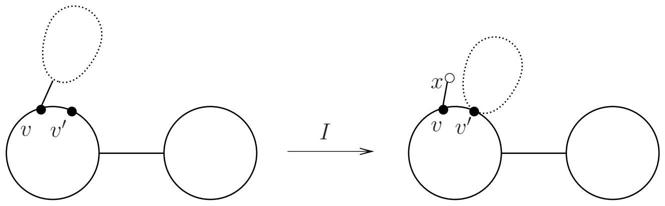

Figure 5. Operation I on bicyclic graphs in type I.

Remark:
(1) For bicyclic graphs in type II, let $v_1 = u_1$. If $v_i = v_1$, without loss of generality, we identify $c_1 \in T_B[v_1]$ and v_2;

(2) For bicyclic graphs in type III, there are $v_1 = u_1, \dots, v_l = u_l$. If $v_i = v_1$ or $v_i = v_l$, without loss of generality, we identify $c_1 \in T_B[v_i]$ and $v_{i+1(\text{mod } p)}$.

For a unicyclic graph U, a similar operation can be defined. We state the following lemma from.[4]

Lemma 0.4. [4] *Let U be a unicyclic graph with $g(U) \geq 4$. Let U^* be the unicyclic graph obtained from U by applying Operation I (replace B by U). If $g(U) \geq 5$, then $W_p(U) < W_p(U^*)$; if $g(U) = 4$, then $W_p(U) \leq W_p(U^*)$.* □

By an analogous analysis as in Lemma 0.4, we can obtain the following observation.

Observation 0.1. *Let B be a bicyclic graph with $p \geq 4$ and $q \geq 4$. Let B^* be the bicyclic graph obtained from B by applying Operation I. Then $W_p(B) \leq W_p(B^*)$.*

Now we define **Operation** II as follows. Let B be a bicyclic graph with $p = 3$, $T_B[v_i]$ be a hanging tree rooted at v_i $(i = 1,2,3)$. Let $v_i c_1 \dots c_{r-1} c_r$ be one of the longest paths from the root v_i to a leaf c_r of the hanging tree $T_B[v_i]$.

For $r \geq 3$, we define a new graph B^* as follows:

$$B^* = \begin{cases} B - c_{r-1}c_r + c_{r-3}c_r, & \text{if } r > 3, \\ B - c_{r-1}c_r + v_i c_r, & \text{if } r = 3. \end{cases} \tag{12}$$

For $r = 2$, the operation differs on the three types of bicyclic graphs.

(1) For bicyclic graphs in type I, we let

$$B^* = \begin{cases} B - c_1c_2 + c_2w_1, & \text{if } v_i = v_1, \\ B - c_1c_2 + c_2v_1, & \text{if } v_i = v_2 \text{ or } v_3, \end{cases} \tag{13}$$

where $w_1 \in N_B(v_i)$ is on the path $v_1 \dots u_1$.

(2) For bicyclic graphs in type II, by considering the value of q, there are two cases.

Case 1. $q \geq 4$. In this case, let

$$B^* = \begin{cases} B - c_1c_2 + c_2v_1, & \text{if } v_i \neq v_1, \\ B - c_1c_2 + c_2u_2, & \text{if } v_i = v_1, \end{cases} \tag{14}$$

where v_i $(i \in \{1,2,3\})$ is the root vertex mentioned above.

Case 2. $q = 3$ and $|V(B)| \geq 9$. Here we let $C_q = v_1v_4v_5v_1$. We define an operation as follows: delete $T_B[v_i] \setminus v_i$ and add a copy of $T_B[v_i]$ to v_j by identifying v_j and v_i' which is a copy of v_i. We call this operation "move $T_B[v_i]$ to v_j". By considering the number of vertices on the cycles of B with hanging trees, there are two subcases.

Subcase 2.1. There is only one vertex v_i $(i \in \{1,2,3,4,5\})$ with a hanging tree. Let $N_B(v_i) = \{c_1^1, \dots, c_1^a\}$, $N_B^2(v_i) = \{c_2^1, \dots, c_2^b\}$.

For the case $v_i = v_1$, we apply operations as follows. If $b \geq 4$, then move c_2^1, c_2^1 to v_2 and c_2^j $(3 \leq j \leq b)$ to v_3; if $b = 3$, then move c_2^1, c_2^2 to v_2 and c_3^2, c_1^1 to v_3; if $b = 2$, then move c_2^1, c_2^2 to v_2 and c_1^1 to v_3; if $b = 1$, then move c_2^1 to v_2 and c_1^1 to v_3. The new graph is denoted by B^*.

For the case $v_i = v_2$, we construct a new graph $B^* = B - c_1c_2 + c_2v_3$.

Subcase 2.2. There are at least two vertices v_s, v_t $(s,t \in \{1,2,3,4,5\})$ with hanging trees. In this subcase, let $B^* = B - c_1c_2 + c_2v_k$, where $v_k \in N_B(v_s) \cap N_B(v_t)$.

(3) For the bicyclic graphs in type III. By considering the value of q, there are two cases.

Case 1. $q \geq 4$. In this case, we can apply Operation I on C_z.

Case 2. $q = 3$ and $|V(B)| \geq 12$. Here let $C_q = v_1v_2v_4v_1$. We can move $T_B[v_4]$ to v_3 to get a new graph B' satisfying $W_p(B') = W_p(B)$. By considering the number of vertices on the cycles of B' with hanging trees, there are two subcases.

Subcase 2.1. There exists only one vertex, say v_i $(i \in \{1,2,3\})$, which has a hanging tree. Firstly, move $T_{B'}[v_i]$ to v_3 (denote the new graph by B''), delete a vertex in $N_{B''}^2(v_3)$ and meanwhile subdivide edge v_1v_4 (denote the new graph by B_1''); secondly, move all the other vertices in $N_{B''}^2(v_3)$ to v_1 (denote the new graph by B'''); thirdly, if $d_{B'''}(v_1) \geq 5$, then just move one pendant vertex of v_1 to v_2; if $d_{B'''}(v_1) = 4$, then move one pendant vertex of v_3 to v_2.

Subcase 2.2. There exist two vertices, say v_i, v_j $(i,j \in \{1,2,3\})$, which have hanging trees. If $i = 1$ and $j = 2$, then move $T_{B'}[v_2]$ to v_3. Now we can only consider the case $i = 1$ and $j = 3$.

If there exists $c_2 \in N_{B'}^2(v_3)$, then delete c_2 and subdivide the edge v_1v_4 (denote the new graph by B''). Now return to the situation in Case 1.

If $N^2(v_3) = \emptyset$ and $d_{B''}(v_3) \geq 4$, then delete a vertex $c_1 \in N_{B''}^2(v_1)$ and subdivide the edge v_1v_4. Now return to Case 1. For the situation that $d_{B''}(v_3) = 3$, delete a vertex $c_2 \in N_{B''}^2(v_1)$ and subdivide the edge v_1v_4, move all pendant vertices in $N_{B''}^2(v_1)$ to v_2, at last move one pendant vertex of v_1 or v_2 to v_3.

Subcase 2.3. There exist three vertices which have hanging trees. By deleting some pendant vertex in $N^2_{B'}(v_i)$, where $i \in \{1,2,3\}$, and meanwhile subdividing the edge v_1v_4, we return to the situation in Case 1.

The final graph obtained after the above operation is denoted by B^*.

Lemma 0.5. *Let B be a bicyclic graph with $p = 3$, $q \geq 3$. Suppose B^* is the graph obtained from B by applying Operation II. Then we have $W_p(B) \leq W_p(B^*)$.*

Proof. Let $v_ic_1c_2\ldots c_r$ be one of the longest paths from root v_i to a leaf c_r in the hanging tree $T_B(v_i)$. By considering the values of r, q and the type of the bicyclic graph, we have the following situations.

According to the above operation and some calculation, we have the following. If $r = 3$, then $W_p(B^*) - W_p(B) \geq (d_B(c_1) - 1) - (d_B(c_1) - 1) = 0$. If $r \geq 4$, then

$$W_p(B^*) - W_p(B) \geq \begin{cases} d_B(c_{r-4}) - 1 \geq 1, & \text{if } r > 4, \\ d_B(v_i) - 1 \geq 1, & \text{if } r = 4. \end{cases} \tag{15}$$

Now suppose $r = 2$.

(1) If B is in type I, then we have

$$W_p(B^*) - W_p(B) \geq \begin{cases} (d_B(v_1) - 1) - (d_B(v_1) - 1) = 0, & \text{if } v_i = v_1, \\ (d_B(v_3) - 2) + (d_B(w_1) - 1) - 1 \geq 0, & \text{if } v_i = v_2, \\ (d_B(v_2) - 2) + (d_B(w_1) - 1) - 1 \geq 0, & \text{if } v_i = v_3. \end{cases} \tag{16}$$

(2) If B is in type II, then by considering the value of p, there are two cases.

Case 1. $q \geq 4$. We have

$$W_p(B^*) - W_p(B) \geq \begin{cases} (d_B(u_2) - 1) - 1 \geq 0, & \text{if } v_i \neq v_1, \\ d_B(u_3) - 1 \geq 1, & \text{if } v_i = v_1. \end{cases} \tag{17}$$

Case 2. $q = 3$ and $|V(B)| \geq 9$. By considering the number of vertices on the cycle of B with hanging trees, there are two subcases.

Subcase 2.1. There is only one vertex v_i $(i \in \{1,2,3,4,5\})$ with a hanging tree.

For the case $v_i = v_1$, we have

$$W_p(B^*) - W_p(B) = \begin{cases} 2[(d_B(v_1) - 2) - (d_B(v_1) - 1)] \\ +(b-2)[(d_B(v_1) - 2 + 2) - (d_B(v_1) - 1)] \geq 0, \text{if } b \geq 4, \\ 3[(d_B(v_1) - 2) - (d_B(v_1) - 1)] + 2 + (d_B(v_1) - 3) \geq 1, \\ \qquad \text{if } b = 3, \\ 2[(d_B(v_1) - 2) - (d_B(v_1) - 1)] + (d_B(v_1) - 3) \geq 0, \\ \qquad \text{if } b = 2, \\ (d_B(v_1) - 2) - (d_B(v_1) - 1) + (d_B(v_1) - 2) \geq 1, \\ \qquad \text{if } b = 1. \end{cases} \tag{18}$$

For the case $v_i = v_2$, we have $W_p(B^*) - W_p(B) = (d_B(v_2) - 2 + 2) - (d_B(v_2) - 1) = 1$.

Subcase 2.2. There are at least two vertices v_s, v_t $(s,t \in \{1,2,3,4,5\})$ with hanging trees. Then we have $W_p(B^*) - W_p(B) \geq (d_B(v_s) - 2) - 1 + (d_B(v_t) - 2) - 1 \geq 0$.

(3) If B is in type III, then there are two cases relying on the value of p.

Case 1. $q \geq 4$. We refer to Observation 0.1.

Case 2. $q = 3$ and $|V(B)| \geq 12$.

It is easy to check that $W_p(B') = W_p(B)$ after moving $T_B[v_4]$ to v_3 .

We only consider Subcase 2.1, since the proof of Subcases 2.2 and 2.3 is analogous to Subcase 2.1. In the first step, after moving $T_{B'}[v_i]$ to v_3, the Wiener polarity index remains the same; after deleting a vertex in $N^2_{B''}(v_3)$ and meanwhile subdividing edge v_1v_4, $W_p(B''_1) - W_p(B'') = [d_{B''}(v_3) - 2] - [d_{B''}(v_3) - 1] = -1$. In the second step (move all the other vertices in $N^2_{B''}(v_3)$ to v_1), $W_p(B''') - W_p(B''_1) = [d_{B''_1}(v_3) - 2 + 1] - [d_{B''_1}(v_3) - 1] = 0$. At last, if $d_{B'''}(v_1) \geq 5$, then after moving one pendant vertex of v_1 to v_2, we have $W_p(B^*) - W_p(B''') = d_{B'''}(v_1) - 4 \geq 1$. Therefore, $W_p(B^*) - W_p(B) \geq 0$ follows. If $d_{B'''}(v_1) = 4$, then after moving one pendant vertex of v_3 to v_2, we have $W_p(B^*) - W_p(B''') = d_{B'''}(v_3) - 3 + 2 - 3 \geq 2$, since $|V(B)| \geq 12$ and $d_{B'''}(v_1) = 4$ implies $d_{B'''}(v_3) \geq 6$. Thus, $W_p(B^*) - W_p(B) \geq 0$ follows. □

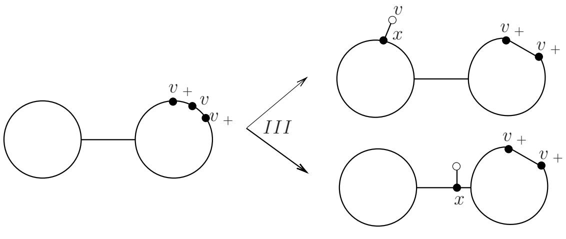

Figure 6. Operation III on bicyclic graphs in type I.

We define **Operation** *III* (see Figure 6) as follows. Let B be a bicyclic graph. If $d_B(v)=2$, then let $B^* = B - vv' - vv'' + v'v'' + vx$, where v', $v'' \in N_B(v)$, $x \in V(B)$. We call such an operation *smooth* v to x.

We define **Operation** *IV* as follows. Let B be a bicyclic graph, where $T_B[v_i]$ $(1 \le i \le p)$ and $T_B[u_j]$ $(1 \le j \le q)$ are both stars. Denote the set of the pendant vertices of v_i (u_j) by V_i (U_j).

For bicyclic graphs in type I, we will take the following two steps (see Figure 7).

Step 1. For C_p and $i \in \{3,\ldots,p-1\}$, if i is odd, then move V_i to v_1 and smooth v_i to v_2; if i is even, then move V_i to v_2 and smooth v_i to v_1. For C_q and $j \in \{3,\ldots,q-1\}$, if j is odd, then move U_j to u_1 and smooth u_j to u_2; if j is even, then move U_j to u_2 and smooth u_j to u_1. Therefore, we obtain a graph $B' = C_{3,3}(s_1,s_2,s_3;t_1,t_2,t_3)$ with a unique path P connecting C_p and C_q. Let the set of hanging leaves of u_1, u_2, u_q be U_1', U_2', U_q', respectively.

Step 2. Let $P = v_1w_1\ldots w_tu_1$, $W_k := T_{B'}[w_k]$ $(1 \le k \le t)$.

If k is odd, then move W_k to v_3 and smooth w_k to v_2; if k is even, then move W_k to v_2 and smooth w_k to v_3.

If t is odd, then move U_1' to v_2, U_2' to v_1, U_q' to v_3; if t is even and $t \ge 2$, then move U_1' to v_3, U_2' to v_1, and U_q' to v_2, respectively; if $t=0$, $|V(B')| \ge 9$ and $d(v_2)=d(v_3)=2$, let $N_{B'}(v_1)=\{a_1,\ldots,a_s\}$ and $N_{B'}(u_1)=\{b_1,\ldots,b_t\}$, then for the situation that $b=1$, move b_1 to v_2 and move a_1 to v_3, for the situation that $b \ge 2$, move b_1 to v_2 and move $b_2,\ldots,b_t$ to v_3; if $t=0$ and $d(v_i)=2$, $d(v_j)>2$ $(i,j \in \{1,2\})$, then move U_1' to v_i, U_2' to v_1, U_q' to v_j, respectively.

Finally, we get a new graph $B'' = C_{3,3}(s_1',s_2',s_3';0,0,0)$ and there is a unique path $P=v_1u_1$ connecting C_p and C_q.

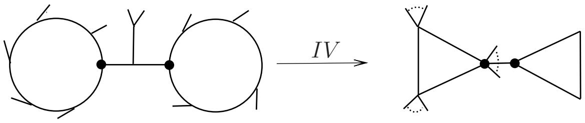

Figure 7. Operation IV on type I.

For bicyclic graphs in type II (see Figure 8), we also give two steps as follows.

Step 1. For C_p and $i \in \{3,\ldots,p-1\}$, if i is odd, then move V_i to v_1 and smooth v_i to v_2; if i is even, then move V_i to v_2 and smooth v_i to v_1. For C_q and $j \in \{3,\ldots,q-1\}$, if j is odd, then move U_j to u_1 and smooth u_j to u_2; if j is even, then move U_j to u_2 and smooth u_j to u_1. Thus we get a graph $B' = C_{3,3}(s_1,s_2,s_3;t_1,t_2,t_3)$ with $s_1=t_1$. Let the set of hanging leaves of u_1, u_2, u_q be U_1', U_2', U_q', respectively.

Step 2. By moving U_2' to v_2, U_q' to v_p, we have $B'' = C_{3,3}(s_1,s_2',s_3';t_1,0,0)$ with $s_1=t_1$.

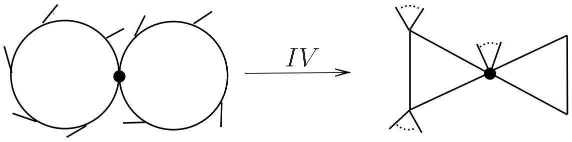

Figure 8. Operation IV on type II.

For bicyclic graphs in type III (see Figure 9), the operation is defined as follows. Recall that we use l (≥ 1) to denote the number of common vertices of C_p and C_q, and without loss of generality, assume $l-2 \le p-2 \le q-2$.

(1) If $p \geq 3$ and $q \geq 4$, then we will take the following three steps.

Step 1. For $i \in \{3,\ldots,p-1\}$, $j \in \{3,\ldots,q-1\}$. If i is odd, then move V_i to v_1; if i is even, then move V_i to v_2; if j is odd, then move U_j to v_1; if j is even, then move U_j to v_2; move U_q to v_p.

Step 2. If $l = 2$ or 3, smooth vertices $v_{l+1},\ldots,v_{p-1}$ to v_1 and v_2 alternately.

If $l \geq 4$, then we first smooth vertices $v_3,\ldots,v_{l-1}$ to v_1 and v_2 alternately; then smooth vertices $v_{l+1},\ldots,v_{p-1}$ to v_1 and v_2 alternately.

After applying this operation, we get a new graph B' with cycles $C_{p'}$, $C_{q'}$ and $C_{z'}$. Let l' be the number of common vertices of $C_{p'}$ and $C_{q'}$, p' ($p' = 3$ *or* 4) be the number of vertices of the smallest cycle of B', then we have $l' = 2$ or $l' = 3$. Now relabel the vertices on $C_{p'}$ and $C_{q'}$ of B', and we have $C_{p'} = v_1 \ldots v_{p'} v_1$ and $C_{q'} = u_1 \ldots u_{q'} u_l$.

Step 3. Considering the value of l', there are two cases.

Case 1. $l' = 2$.

We just smooth $u_{l+2} \ldots u_{q'-2}$ to v_1 and v_2 alternately, and smooth $u_{q'-1}$ to v'_p. The new graph obtained is denoted by $B^* = C_{3,4}(s_1,s_2,s_3;s_1,s_2,0,0)$.

Case 2. $l' = 3$, $|V(B')| \geq 6$ and $B' = C_{4,q}(s_1,s_2,0,s_4;t_1,t_2,0,\ldots,0)$ with $s_1 = t_1$.

Let $B'' = B' - v_3v_4 + v_2v_4$. If $q' \geq 5$, then smooth v_3 to vertex v_1, smooth $u_5,\ldots,u_{q'-2}$ to v_1 and v_2 alternately and smooth $u_{q'-1}$ to v_4; if $q' = 4$ and $d_{B''}(v_4) \geq 3$, then we do nothing; if $q' = 4$ and $d_{B''}(v_4) = 2$, then move the pendant vertices of v_2 to v_4.

Finally, we get the desired graph $B^* = C_{3,4}(s'_1,s'_2,s'_3;s'_1,s'_2,0,0)$.

(2) If $p = 3$ and $q = 3$, by Operation *II* on B and its resultant graphs repeatedly, we have a new graph $B' = C_{3,3}(s'_1,s'_2,s'_3;s'_1,s'_2,t'_3)$. Move the pendant vertices of u_3 to v_3, we obtain $B'' = C_{3,3}(s''_1,s''_2,s''_3;s''_1,s''_2,0)$.

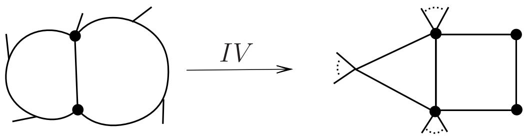

Figure 9. Operation IV on type III.

Lemma 0.6. *By applying Operation IV to the corresponding bicyclic graphs, the Wiener polarity index is not decreasing.*

Proof. Note that there are mainly two steps "move" and "smooth". We want to prove that each step ensures that the value of the Wiener polarity index is not decreasing. Here we denote the final bicyclic graph by B^*.

There are three kinds of unordered vertices pair $\{u,v\}$ such that $d_B(u,v) = 3$ on $B = C_{p,q}(s_1,\ldots,s_p;t_1,\ldots,t_q)$: u and v are both pendant vertices; u and v are both on the cycle of B; u is a pendant vertex and v is on the cycle of B.

Since we move V_i, V_{i+1} $(i \in \{3,\ldots,p-1\})$ (or U_j, U_{j+1} $(j \in \{3,\ldots,q-1\})$ to two adjacent vertices alternately, we keep the unordered vertices pair $\{u,v\}$, where u and v are both pendant vertices. When we apply Operation *III* ("*smooth*"), in the same way, we smooth the vertices to two adjacent vertices alternately which remains the unordered vertices pair $\{u,v\}$ of the second kind. At last, we only need to consider the unordered vertices pair $\{u,v\}$ of the third kind. Since there are at most three vertices v_1, v_2 and v_p on B^* with pendant vertices, then if the unordered vertices pair $\{u,v\}$ in the original graph is composed by $u \in V_i$ $(i \in \{1,2,\ldots,p\})$ (or U_j $(j \in \{1,2,\ldots,q\})$) and $v \in V(C_p) \cup V(C_q)$, then after such operation it can be replaced by u' (a pendant vertex of $v_k \in V(B^*)$ $(k \in \{1,2,p\})$) and $v' \in N^2_{B^*}(v_k)$.

As to the operation on bicyclic graphs in type *I* which is mentioned in Step 2, the proof is just analogous.

Combining the three situations above, we complete the proof. □

Since our goal is to characterize the bicyclic graphs of given order maximizing the Wiener polarity index, then after the introduction of all these operations on bicyclic graphs, we will show the procedure step by step to reach the desired graph. Meanwhile, according to the lemma above, we claim that each step ensures the Wiener polarity index of the new graph being not decreasing.

Let B_1 be a bicyclic graph of type *I* and $|V(B_1)| \geq 15$.

(1) If $p \geq 4$ and $q \geq 4$, then we will do by two steps.

Step 1. By applying Operation *I* on B_1 and its resultant graphs repeatedly, we get a graph $B_1^1 = C_{p,q}(s_1,\ldots,s_p;t_1,\ldots,t_q)$ satisfying that $W_p(B_1^1) \geq W_p(B_1)$.

Step 2. By Operation IV on B_1^1, We get the desired graph $B_1^2 = C_{3,3}(s_1,s_2,s_3;0,0,0)$ with $P = u_1v_1$. Moreover, $W_p(B_1^2) \geq W_p(B_1^1)$.

(2) If $p = 3$ and $q \geq 4$, then we will take the following two steps.

Step 1. By Operation II on $C_p = v_1v_2v_3v_1$ and its resultant graphs repeatedly, Operation I on $C_q = u_1u_2\dots u_qu_1$ and its resultant graphs repeatedly, we get graph B_1^1. Observe that $T_{B_1^1}(v_i)$ $(1 \leq i \leq p)$ and $T_{B_1^1}(u_j)$ $(1 \leq j \leq q)$ are both stars.

Step 2. By applying Operation IV on B_1^1, we reach $B_1^2 = C_{3,3}(s_1,s_2,s_3;0,0,0)$ with $P = u_1v_1$.

(3) Suppose $p = 3$, $q = 3$. Applying Operations II and IV on B_1 and its resultant graphs repeatedly, we obtain the desired graph $B_1^1 = C_{3,3}(s_1,s_2,s_3;0,0,0)$ with $P = u_1v_1$.

Finally, by the steps above, the maximum Wiener polarity index of bicyclic graphs in type I is determined. We denote the desired graph by B_1^*, and clearly that the unique path connecting C_p and C_q is $P = v_1u_1$.

Let $C'_{3,3}(s_1,s_2,s_3;t_1,t_2,t_3)$ be the bicyclic graph of type I, where $P = v_1u_1$ and $s_1+s_2+s_3+t_1+t_2+t_3 = n-6$. Especially, we denote this kind of graphs by $C^*_{3,3}$, if $t_1 = t_2 = t_3 = 0$, $0 \leq s_1 - s_i \leq 2$ $(i = 2,3)$, $|s_2 - s_3| \leq 1$. For a graph $G = (V,E)$ and $P_l = v_1v_2\dots v_{l+1}$, we can construct a new graph H by identifying v_1 with $v \in G$, denoted by $H := G + P_l$, and we say P_l is *incident* to vertex v.

Theorem 0.1. *Let B_1 be a bicyclic graph in type I and $|V(B_1)| = n$ (≥ 6), B_1^* be the desired graph attaining the maximum Wiener polarity index.*

(1) If $n = 6$, then $B_1^* = C'_{3,3}(0,0,0;0,0,0)$, and $W_p(B_1) = W_p(B_1^*) = 4$;

(2) If $n = 7$, then $B_1^* \cong C'_{3,3}(1,0,0;0,0,0)$, and $W_p(B_1) \leq W_p(B_1^*) = 6$;

(3) If $n = 8$, then $B_1^* \cong C'_{3,3}(1,0,0;1,0,0)$, $C'_{3,3}(1,0,0;0,0,0) + P_1$, where P_1 is incident to the pendant vertex of v_1, and $W_p(B_1) \leq W_p(B_1^*) = 9$;

(4) If $n = 9$, then $B_1^* \cong C'_{3,3}(2,0,0;1,0,0)$, $C'_{3,3}(1,0,0;1,0,0) + P_1$, where the path P_1 is incident to the pendant vertex of v_1, $C'_{3,3}(2,0,0;0,0,0) + P_1$, where the path P_1 is incident to one pendant vertex of v_1, $C'_{3,3}(1,0,0;0,0,0) + P_1 + P_1$, where the two paths P_1 are incident to the pendant vertex of v_1, and $W_p(B_1) \leq W_p(B_1^*) = 12$;

(5) If $n = 10$, then $B_1^* \cong C'_{3,3}(2,0,0;2,0,0)$, $C'_{3,3}(2,0,0;1,0,0) + P_1$, where the path P_1 is incident to one pendant vertex of v_1, $C'_{3,3}(2,0,0;0,0,0) + P_1 + P_1$, where the two paths P_1 are incident to the pendant vertices of v_1, and $W_p(B_1) \leq W_p(B_1^*) = 16$;

(6) If $n = 11$, then $B_1^* \cong C'_{3,3}(3,0,0;2,0,0)$, $C'_{3,3}(2,0,0;2,0,0) + P_1$, where the path P_1 is incident to one pendant vertex of v_1, $C'_{3,3}(2,0,0;1,0,0) + P_1 + P_1$, where the two paths P_1 are incident to the pendant vertices of v_1, $C'_{3,3}(2,0,0;0,0,0) + P_1 + P_1 + P_1$, where the three paths P_1 are incident to the pendant vertices of v_1, and $W_p(B_1) \leq W_p(B_1^*) = 20$;

(7) If $n = 12$, then $B_1^* \cong C'_{3,3}(3,0,0;3,0,0)$, $C'_{3,3}(3,0,0;2,0,0) + P_1$, where P_1 is incident to one pendant vertex of v_1, $C'_{3,3}(3,0,0;1,0,0) + P_1 + P_1$, where the two paths P_1 are incident to the pendant vertices of v_1, $C'_{3,3}(3,0,0;0,0,0) + P_1 + P_1 + P_1$, where the three paths P_1 are incident to the pendant vertices of v_1, and $W_p(B_1) \leq W_p(B_1^*) = 25$;

(8) If $n = 13$, then $B_1^* \cong C^*_{3,3}$, $C'_{3,3}(4,0,0;3,0,0)$, $C'_{3,3}(3,0,0;3,0,0) + P_1$, where P_1 is incident to one pendent vertex of v_1, $C'_{3,3}(4,0,0;2,0,0) + P_1$, where P_1 is incident to one pendant vertex of v_1, $C'_{3,3}(4,0,0;1,0,0) + P_1 + P_1$, where the two paths P_1 are incident to the pendant vertices of v_1, $C'_{3,3}(3,0,0;2,0,0) + P_1 + P_1$, where the two paths P_1 are incident to the pendant vertices of v_1, $C'_{3,3}(4,0,0;0,0,0) + P_1 + P_1 + P_1$, where the three paths P_1 are incident to the pendant vertices of v_1, $C'_{3,3}(3,0,0;1,0,0) + P_1 + P_1 + P_1$, where the three paths P_1 are incident to the pendant vertices of v_1, $C'_{3,3}(4,0,0;0,0,0) + P_1 + P_1 + P_1 + P_1$, where the four paths P_1 are incident to the pendant vertices of v_1, and $W_p(B_1) \leq W_p(B_1^*) = 30$;

(9) If $n = 14$, then $B_1^* \cong C^*_{3,3}$, $C'_{3,3}(4,0,0;4,0,0)$, $C'_{3,3}(4,0,0;3,0,0) + P_1$, where P_1 is incident to one pendent vertex of v_1, $C'_{3,3}(4,0,0;2,0,0) + P_1 + P_1$, where the two paths P_1 are incident to the pendant vertices of v_1, $C'_{3,3}(4,0,0;1,0,0) + P_1 + P_1 + P_1$, where the three paths P_1 are incident to the pendant vertices of v_1, $C'_{3,3}(4,0,0;0,0,0) + P_1 + P_1 + P_1 + P_1$, where the four paths P_1 are incident to the pendant vertices of v_1, and $W_p(B_1) \leq W_p(B_1^*) = 36$;

(10) If $n \geq 15$, then $B_1^* \cong C^*_{3,3}$, and $W_p(B_1) \leq W_p(B_1^*)$.

Proof. If $6 \leq n \leq 14$, then we can get the desired result mentioned above by directly computations.

If $n \geq 15$, we can get $C'_{3,3}(s_1,s_2,s_3;0,0,0)$ by the operation mentioned in the corresponding steps. Now we want to prove that $B_1^* = C^*_{3,3}$. Assume to the contrary that there exists $i \in \{2,3\}$, such that $s_1 - s_i > 2$ or $s_1 - s_i < 0$.

If $s_1 - s_i > 2$, without loss of generality, suppose $i = 2$. By moving a pendant edge of v_1 to v_2, we obtain a new graph, denoted by B'_1, and we have $W_p(B'_1) - W_p(B_1^*) = (s_1 - 1 + 1 + s_3) - (s_3 + s_2 + 2) > 0$, a contradiction.

If $s_1 - s_i < 0$, without loss of generality, suppose $i = 2$. By moving a pendant edge of v_1 to v_2, we obtain a new graph, denoted by B''_1, and we have $W_p(B''_1) - W_p(B_1^*) = (s_2 - 1 + s_3 + 2) - (s_1 + 1 + s_3) = s_2 - s_1 > 0$, a contradiction.

By a similar discussion, $|s_2 - s_3| \leq 1$ follows.

Consequently, we have $B_1^* = C^*_{3,3}$. □

Let B_2 be a bicyclic graph of type II $(v_1 = u_1)$ and $|V(B_2)| \geq 9$.

(1) If $p \geq 4$ and $q \geq 4$, then we can obtain the desired graph by two steps.

Step 1. By applying Operation I on B_2 and its resultant graphs repeatedly, we obtain $B_2^1 = C_{p,q}(s_1,\ldots,s_p;t_1,\ldots,t_q)$ with $s_1 = t_1$.

Step 2. By Operation IV on B_2^1, we get $B_2^2 = C_{3,3}(s_1',s_2',s_3';t_1',0,0)$ with $s_1' = t_1'$ and $W_p(B_2^2) \geq W_p(B_2^1)$.

(2) If $p = 3$ and $q \geq 4$, take the following two steps.

Step 1. We first apply Operation I on C_q, II on C_p and its resultant graphs repeatedly, we obtain a new graph $B_2^1 = C_{3,q}(s_1,s_2,s_3;t_1,t_2,\ldots,t_q)$ with $s_1 = t_1$.

Step 2. By Operation IV on B_2^1, we obtain $B_2^2 = C_{3,3}(s_1',s_2',s_3';t_1',0,0)$ with $s_1' = t_1'$.

(3) If $p = 3$, $q = 3$.

Apply the corresponding operation in operation II and IV to B_2 and its resultant graphs, finally we reach the desired graph $B_2^1 = C_{3,3}(s_1',s_2',s_3';t_1',0,0)$ with $s_1' = t_1'$.

Finally, by the steps above, the maximum Wiener polarity index of bicyclic graphs in type II is determined. We denote the desired graph by B_2^*.

Let $C_{3,3}''(s_1,s_2,s_3;t_1,t_2,t_3)$ be the bicyclic graph in type II, where $s_1+s_2+s_3+t_1+t_2+t_3 = n-5$, and $s_1 = t_1$. When n is large enough, it can be easily checked that the graph maximizing the Wiener polarity index is $B_2^* = C_{3,3}''(s_1,s_2,s_3;s_1,0,0)$.

Theorem 0.2. *Let B_2 be a bicyclic graph in type II and $|V(B_2)| = n$ (≥ 5), B_2^* be the desired graph attaining the maximum Wiener polarity index.*

(1) If $n = 5$, then $B_2^* = B_2 = C_{3,3}''(0,0,0;0,0,0)$, and $W_p(B_2) = 0$;

(2) If $n = 6$, then $B_2^* \cong C_{3,3}''(0,1,0;0,0,0)$, $C_{3,4}''(0,0,0;0,0,0,0)$, and $W_p(B_2) \leq W_p(B_2^*) = 2$;

(3) If $n = 7$, then $B_2^* \cong C_{3,3}''(0,1,1;0,0,0)$, $C_{3,4}''(0,0,0;0,1,0,0)$, and $W_p(B_2) \leq W_p(B_2^*) = 5$;

(4) If $n = 8$, then $B_2^* \cong C_{3,3}''(0,1,2;0,0,0)$, $C_{3,4}''(0,0,0;0,1,0,1)$, and $W_p(B_2) \leq W_p(B_2^*) = 8$;

(5) For $n \geq 9$, let $s_1+s_2+s_3 = 3k+r$ $(r \in \{0,1,2\})$.

If $r = 0$, then $B_2^* \cong C_{3,3}''(k-2,k+1,k+1;k-2,0,0)$, $C_{3,3}''(k-1,k,k+1;k-1,0,0)$, and $W_p(B_2) \leq W_p(B_2^*) = 3k^2+4k+1$;

If $r = 1$, then $B_2^* \cong C_{3,3}''(k-1,k+1,k+1;k-1,0,0)$, and $W_p(B_2) \leq W_p(B_2^*) = 3k^2+6k+3$;

If $r = 2$, then $B_2^* \cong C_{3,3}''(k-1,k+1,k+2;k-1,0,0)$, and $W_p(B_2) \leq W_p(B_2^*) = 3k^2+8k+5$.

Proof. If $5 \leq n \leq 8$, then we can get the desired result mentioned above by directly computation.

Now suppose $n \geq 9$. Let U be the unicyclic graph obtained from $B_2^* = C_{3,3}''(s_1,s_2,s_3;s_1,0,0)$ by deleting edge u_1u_2, where $u_1u_2 \in E(B_2^*)$ is an edge of C_q. Note that $W_p(U) = W_p(B_2^*)$, we can determine $W_p(B_2^*)$ in the perspective of $W_p(U)$.

Denote the cycle of U by $C = v_1v_2v_3v_1$. Let a_i be the number of the pendant edges of $v_i (1 \leq i \leq 3)$. Now we claim that $|a_i - a_j| \leq 1$ $(1 \leq i,j \leq 3)$.

By contradiction. Without loss of generality, assume that $a_1 - a_2 > 1$, then by moving a pendant edge of v_1 to v_2, we have a new graph denoted by U', and $W_p(U') - W_p(U) = (a_1 - 1 + a_3) - (a_2 + a_3) = a_1 - a_2 > 0$, a contradiction. Thus, $|a_i - a_j| \leq 1$ $(1 \leq i,j \leq 3)$ is attained.

Now we apply the result to B_2^*. By some corresponding computations, (5) follows. □

Let B_3 be a bicyclic graph of type III and $|V(B_3)| \geq 12$.

(1) If $p \geq 4$ and $q \geq 4$, then we can obtain the desired graph as follows.

Step 1. By applying Operation I on B_3 and its resultant graphs repeatedly, we obtain a new graph B_3^1. Observe that $T_{B_3^1}(v_i)$ $(1 \leq i \leq p)$ and $T_{B_3^1}(u_j)$ $(1 \leq j \leq q)$ are both stars.

Step 2. Applying Operation IV on B_3^1. We have $B_3^2 = C_{3,4}(s_1,s_2,s_3;t_1,t_2,0,0)$ with $s_1 = t_1$, $s_2 = t_2$.

(2) If $p = 3$, $q \geq 4$, then we take the following two steps.

Step 1. By Operation I on C_q, II on C_p and its resultant graphs repeatedly, we obtain $B_3^1 = C_{3,q}(s_1,s_2,s_3;t_1,t_2,\ldots,t_q)$ with $s_1 = t_1$, $s_2 = t_2$.

Step 2. Applying Operation IV on B_3^1, we get the desired graph $B_3^2 = C_{3,4}(s_1',s_2',s_3';t_1',t_2',0,0)$ with $s_1' = t_1'$, $s_2' = t_2'$.

(3) Suppose $p = 3$, $q = 3$ and $|V(B_3)| \geq 12$. Applying the Operations II and IV on B_3 and its resultant graphs repeatedly, we reach the desired graph $B_3^2 = C_{3,3}(s_1',s_2',s_3';t_1',t_2',0)$ with $s_1' = t_1'$, $s_2' = t_2'$.

Finally, by the steps above, the maximum Wiener polarity index of bicyclic graphs in type III is determined. We denote the desired graph by B_3^*.

Let $C_{3,3}'''(s_1,s_2,s_3;t_1,t_2,t_3)$ be the bicyclic graph in type III, where $s_1+s_2+s_3+t_1+t_2+t_3 = n-4$, $s_1 = t_1$, $s_2 = t_1$ and $l = 1$. Let $C_{3,4}'''(s_1,s_2,s_3;t_1,t_2,t_3,t_4)$ be the bicyclic graph in type III, where $s_1+s_2+s_3+t_1+t_2+t_3+t_4 = n-5$, $s_1 = t_1$, $s_2 = t_1$ and $l = 1$. When n is large enough, it can be checked that the graph maximizing the Wiener polarity index is $B_3^* = C_{3,4}'''(s_1,s_2,s_3;s_1,s_2,0,0)$.

Theorem 0.3. *Let B_3 be a bicyclic graph in type III and $|V(B_3)| = n$ (≥ 4), B_3^* be the desired graph attaining the maximum Wiener polarity index.*

(1) If $n = 4$, then $B_3^* = B_3 = C'''_{3,3}(0,0,0;0,0,0)$, and $W_p(B_3) = 0$;

(2) If $n = 5$, then $B_3^* \cong C'''_{3,3}(0,0,1;0,0,0)$, and $W_p(B_3) \leq W_p(B_3^*) = 1$;

(3) If $n = 6$, then $B_3^* \cong C'''_{3,3}(0,0,0;0,0,0) + P_2$, where P_2 is incident to vertex v_1 or v_3, and $W_p(B_3) \leq W_p(B_3^*) = 3$;

(4) If $n = 7$, then $B_3^* \cong C'''_{3,3}(1,0,0;1,0,0) + P_1 + P_1$, where the two paths P_1 are incident to the pendant vertex of v_1, and $W_p(B_3) \leq W_p(B_3^*) = 6$;

(5) If $n = 8$, then $B_3^* \cong C'''_{3,3}(1,0,0;1,0,0) + P_1 + P_1 + P_1$, where the three paths P_1 are incident to the pendant vertex of v_1, and $W_p(B_3) \leq W_p(B_3^*) = 9$;

(6) If $n = 9$, then $B_3^* \cong C'''_{3,3}(1,0,0;1,0,0) + P_1 + P_1 + P_1 + P_1$, where the four paths P_1 are incident to the pendant vertex of v_1, $C'''_{3,3}(2,0,0;2,0,0) + P_1 + P_1 + P_1$, where the three paths P_1 are incident to the pendant vertices of v_1, and $W_p(B_3) \leq W_p(B_3^*) = 12$;

(7) If $n = 10$, then $B_3^* \cong C'''_{3,3}(2,0,0;2,0,0) + P_1 + P_1 + P_1 + P_1$, where the four paths P_1 are incident to the pendant vertives of v_1, and $W_p(B_3) \leq W_p(B_3^*) = 16$;

(8) If $n = 11$, then $B_3^* \cong C'''_{3,3}(2,0,0;2,0,0) + P_1 + P_1 + P_1 + P_1 + P_1$, where the five paths P_1 are incident to the pendant vertices of v_1, $C'''_{3,3}(3,0,0;3,0,0) + P_1 + P_1 + P_1 + P_1$, where the four paths P_1 are incident to the pendant vertices of v_1, $C'''_{3,4}(2,2,2;2,2,0,0)$, $C'''_{3,4}(1,2,3;1,2,0,0)$, and $W_p(B_3) \leq W_p(B_3^*) = 20$;

(9) For $n \geq 12$, let $s_1 + s_2 + s_3 = 3k + r$ $(r \in \{0,1,2\})$.

If $r = 0$, then $B_3^* \cong C'''_{3,4}(k-1,k-1,k+2;k-1,k-1,0,0)$, $C'''_{3,4}(k-1,k,k+1;k-1,k,0,0)$, and $W_p(B_3) \leq W_p(B_3^*) = 3k^2 + 2k + 1$;

If $r = 1$, then $B_3^* \cong C'''_{3,4}(k,k,k+1;k,k,0,0)$, $C'''_{3,4}(k-1,k,k+2;k-1,k,0,0)$, and $W_p(B_2) \leq W_p(B_2^*) = 3k^2 + 4k + 2$;

If $r = 2$, then $B_3^* \cong C'''_{3,4}(k,k,k+2;k,k,0,0)$, and $W_p(B_3) \leq W_p(B_3^*) = 3k^2 + 6k + 4$.

Proof. If $4 \leq n \leq 11$, then we can get the desired result mentioned above by directly computation.

If $n \geq 12$, we can get $C'_{3,4}(s_1,s_2,s_3;s_1,s_2,0,0)$ by the operation mentioned in the corresponding steps. Now we claim that $1 \leq s_3 - s_i \leq 3 (1 \leq i \leq 2)$ and $|s_1 - s_2| \leq 1$. We prove this by contradiction.

If $s_3 - s_i = 0$ $(1 \leq i \leq 2)$, then by moving a pendant edge of v_1 to v_3, we obtain a new graph $B^{'}$, and we have $W_p(B') - W_p(B_3^*) = (s_2 + 1 + s_1 - 1 + 1) - (s_3 + s_2) = 1 > 0$, a contradiction.

If $s_3 - s_i > 4$ $(1 \leq i \leq 2)$, without loss of generality, suppose $i = 1$, then by moving a pendant edge of v_3 to v_1, we obtain a new graph B'', and we have $W_p(B'') - W_p(B_3^*) = (s_3 - 1 + s_2) - (s_1 + 1 + s_2 + 1) = s_3 - s_1 - 3 > 0$, a contradiction.

If $|s_1 - s_2| > 1$, without loss of generality, suppose $s_1 - s_2 > 1$, then by moving a pendant edge of v_1 to v_2, we obtain a new graph B''', and we have $W_p(B''') - W_p(B_3^*) = (s_3 + s_1 - 1) - (s_3 + s_2) = s_1 - s_2 - 1 > 0$, a contradiction.

Hence, we complete the proof of the claim.

By the claim and some computations, (9) follows. □

Now combining Theorems 1.1, 1.2 and 1.3, the maximum polarity index of bicyclic graphs is determined, which is shown in the following theorem.

Theorem 0.4. *Let B be a bicyclic graph of order n (≥ 4), B^* be the bicyclic graph with the maximum polarity index among all bicyclic graphs.*

(1) If $n = 4$, then $B^* = B = C'''_{3,3}(0,0,0;0,0,0)$, and $W_p(B_3) = 0$;

(2) If $n = 5$, then $B^* \cong C'''_{3,3}(0,0,1;0,0,0)$, and $W_p(B) \leq W_p(B^*) = 1$;

(3) If $n = 6$, then $B^* \cong C'_{3,3}(0,0,0;0,0,0)$, and $W_p(B) \leq W_p(B^*) = 4$;

(4) If $n = 7$, then $B_1^* \cong C'_{3,3}(1,0,0;0,0,0)$, $C'''_{3,3}(1,0,0;1,0,0) + P_1 + P_1$, where the two paths P_1 are incident to the pendant vertex of v_1 and $W_p(B_1) \leq W_p(B_1^*) = 6$;;

(5) If $n = 8$, then $B^* \cong C'_{3,3}(1,0,0;1,0,0)$, $C'_{3,3}(1,0,0;0,0,0) + P_1$, where P_1 is incident to one pendant vertex of v_1, $C'''_{3,3}(1,0,0;1,0,0) + P_1 + P_1 + P_1$, where the three paths P_1 are incident to the pendant vertex of v_1, and $W_p(B) \leq W_p(B^*) = 9$;

(6) If $n = 9$, then $B^* \cong C'_{3,3}(2,0,0;1,0,0)$, $C'_{3,3}(1,0,0;1,0,0) + P_1$, where the path P_1 is incident to the pendant vertex of v_1, $C'_{3,3}(2,0,0;0,0,0) + P_1$, where the path P_1 is incident to one pendant vertex of v_1, $C'_{3,3}(1,0,0;0,0,0) + P_1 + P_1$, where the two paths P_1 are incident to the pendant vertex of v_1, $C''_{3,3}(0,2,2;0,0,0)$, $C'''_{3,3}(1,0,0;1,0,0) + P_1 + P_1 + P_1 + P_1$, where the four paths P_1 are incident to the pendant vertex of v_1, $C'''_{3,3}(2,0,0;2,0,0) + P_1 + P_1 + P_1$, where the three paths P_1 are incident to the pendant vertices of v_1, and $W_p(B_1) \leq W_p(B_1^*) = 12$;

(7) If $n = 10$, then $B^* \cong C'_{3,3}(2,0,0;2,0,0)$, $C'_{3,3}(2,0,0;1,0,0) + P_1$, where the path P_1 is incident to one pendant vertex of v_1, $C'_{3,3}(2,0,0;0,0,0) + P_1 + P_1$, where the two paths P_1 are incident to the pendant vertices of v_1, $C''_{3,3}(0,2,3;0,0,0)$,

$C'''_{3,3}(2,0,0;2,0,0)+P_1+P_1+P_1+P_1$, *where the four paths* P_1 *are incident to the pendant vertices of* v_1, *and* $W_p(B_1) \leq W_p(B_1^*) = 16$*;*

(8) For $n \geq 11$, *let* $s_1+s_2+s_3 = 3k+r$ $(r \in \{0,1,2\})$.

If $r=0$, *then* $B^* \cong C''_{3,3}(k-2,k+1,k+1;k-2,0,0), C''_{3,3}(k-1,k,k+1;k-1,0,0)$, *and* $W_p(B) \leq W_p(B^*) = 3k^2+4k+1$*;*

If $r=1$, *then* $B^* \cong C''_{3,3}(k-1,k+1,k+1;k-1,0,0)$, *and* $W_p(B) \leq W_p(B^*) = 3k^2+6k+3$*;*

If $r=2$, *then* $B^* \cong C''_{3,3}(k-1,k+1,k+2;k-1,0,0)$, *and* $W_p(B) \leq W_p(B^*) = 3k^2+8k+5$. □

1References

1. Bondy, J. A. & Murty, U. S. R. (eds.) *Graph Theory* (Springer–Verlag, 2008). Berlin.

2. Du, W., Li, X. & Shi, Y. Algorithms and extremal problem on wiener polarity index. *MATCH Commum. Math. Comput. Chem.* **62**, 235–244 (2009).

3. Liu, M. & Liu, B. On the wiener polarity index. *MATCH Commun. Math. Comput. Chem.* **66**, 293–304 (2011).

4. Hou, H., Liu, B. & Huang, Y. On the wiener polarity index of unicyclic graphs. *Appl. Math. Comput.* **218**, 10149–10157 (2012).
